# Research on floating object classification algorithm based on convolutional neural network

**DOI:** 10.1038/s41598-024-83543-9

**Published:** 2024-12-30

**Authors:** Jikai Yang, Zihan Li, Ziyan Gu, Wei Li

**Affiliations:** School of Naval Architecture and Ocean Engineering, Huazhong University of Science and Technology, Wuhan, 430074 China

**Keywords:** Convolutional Neural Network, Unmanned Boat, Surface Floating Debris, Image Recognition, Data Augmentation, Ecology, Environmental sciences, Engineering, Mathematics and computing, Computer science, Information technology

## Abstract

With the advancement of artificial intelligence technology, unmanned boats utilizing deep learning models have shown significant potential in water surface garbage classification. This study employs Convolutional Neural Network (CNN) to extract features of water surface floating objects and constructs the VGG16-15 model based on the VGG-16 architecture, capable of identifying 15 common types of water surface floatables. A garbage classification dataset was curated to obtain 5707 images belonging to 15 categories, which were then split into training and validation sets in a 4:1 ratio. Customized improvements were made on the base VGG-16 model, including adjusting the neural network structure to suit 15 floating object categories, applying learning rate decay and early stopping strategies for model optimization, and using data augmentation to enhance model generalization. By tweaking certain parameters, the study analyzed the impact of the number of epochs and batch sizes on the model’s classification effectiveness. The results show that the model achieves the best performance with 20 epochs and a batch size of 64, reaching a recognition accuracy of 93.86%. This is a 10.09% improvement over the traditional VGG-16 model and a 4.91% increase compared to the model without data augmentation, demonstrating the effectiveness of model improvements and data augmentation in enhancing image recognition capabilities. Additionally, the few-shot test demonstrates the fine-tuned model’s improved generalization capability. This research illustrates the applicability of transfer learning in the task of water surface garbage classification and provides technical support for the application of unmanned boats in environmental protection.

## Introduction

With the increasing impact of human activities on the environment, floating trash on water surfaces has become a global environmental issue. This trash not only damages aquatic ecosystems but also severely pollutes water quality, threatening both aquatic life and human health^1,2^. Therefore, effective monitoring and classification of water surface trash have become essential tasks in environmental protection^3,4^. With the rapid development of artificial intelligence^5,6^, especially the breakthroughs in deep learning for image processing, new opportunities have arisen for the automatic identification and classification of floating trash on water surfaces^7^. In recent years, Convolutional Neural Networks (CNNs) have achieved remarkable progress in image recognition^8–10^, and many studies have successfully applied CNNs to tasks such as natural image processing, object detection, and classification^11^. Notably, the VGG-16 model, with its deep structure and excellent feature extraction capabilities^12^, has demonstrated outstanding performance across various image processing domains. Additionally, new optimization algorithms like the Puma Optimizer^13^, which enhances feature selection and model optimization, have introduced innovative approaches to classification model development, showing strong performance in complex environments^14^. In urban management, comparing different deep learning architectures has shown that adjusting model structures for specific tasks can significantly improve accuracy and efficiency^15^, offering valuable insights for applications such as environmental monitoring^16^. In recent years, unmanned boats have become essential tools for water environment monitoring and pollution management^17,18^. Equipped with deep learning models, these boats can perform real-time classification and detection of floating objects on the water surface, providing an efficient solution for environmental protection^19^. This deep learning-based recognition system not only enhances the intelligence level of unmanned boats in complex environments but also improves the accuracy and efficiency of floating trash classification^20–22^. However, while research indicates that the application of deep learning on unmanned platforms continues to expand, stable and efficient deployment in real-world operations requires further model optimization to meet the hardware constraints of embedded systems^23^. This study aims to explore an improved VGG-16 model combined with a transfer learning approach. First, an image dataset containing 15 common categories of floating objects on water surfaces was constructed. Subsequently, the VGG-16 model was adapted to better fit the characteristics of water surface debris, with data augmentation techniques applied to enhance model generalization. Additionally, the effects of different learning rates, epochs, and batch sizes on model performance were analyzed to determine the optimal training strategy. The objective of this study is to improve the classification accuracy of floating debris and provide technical support for the application of unmanned boats in environmental protection.

## Model introduction and improvement

### Dataset preparation and preprocessing

In order to establish an efficient model for water surface floating trash recognition, and the model is required to have a stable correct rate guarantee in practical applications, it is necessary to collect and prepare an adequate dataset. In the process of dataset construction, we systematically analyzed the types of floating objects on water surfaces to ensure data representativeness and model applicability. Floating objects on water are diverse and can be categorized into natural and man-made objects. To achieve effective classification and ensure the model’s applicability in various practical scenarios, we selected 15 representative categories for this study. These categories not only cover most common floating debris, such as leaves and branches under natural degradation, plastic bags and foam boards among plastic products, and textiles like clothing and towels, but also include other common pollutants such as glass bottles and cigarette butts. This categorization is based on the distribution of floating objects across different water environments, covering the majority of main components of surface waste. The selection of the 15 floating object categories was based on both their prevalence in real-world environments and their significance in environmental monitoring and waste management. Floating debris on water surfaces poses significant threats to aquatic ecosystems and water quality, making the identification of key categories essential for effective monitoring and cleanup. These 15 categories were chosen to cover a wide range of pollutants commonly found in urban and natural water bodies. Categories such as plastic bags, foam panels, and bottles represent common and highly persistent pollutants that significantly affect water quality. Items like branches and leaves, while naturally occurring, also impact water quality and aquatic life as they decompose. Additionally, this selection reflects the diverse nature of floating debris, from synthetic materials to biodegradable organic matter, ensuring that the model is applicable to various environmental conditions. In the context of unmanned boats, these categories enable the development of a system capable of addressing both frequently encountered and less common debris, thereby enhancing the system’s robustness. By focusing on these 15 representative categories, the model is not only aligned with the most prevalent real-world scenarios but also provides a scalable framework that can be expanded to accommodate additional categories as required for specific environmental monitoring tasks. Selecting these 15 categories ensures strong representativeness, providing scientific and systematic support for the dataset while also ensuring the model’s broad applicability in different environments, thus avoiding recognition errors due to over- or under-categorization. This selection is therefore both reasonable and essential.

To ensure balance across categories, we paid special attention to the distribution of samples in the dataset. We conducted an analysis of the number of samples in both the training and validation sets for each category, ensuring that the dataset was as balanced as possible. In cases where there was some imbalance across categories, we applied targeted data augmentation techniques, particularly focusing on increasing the variety of samples for underrepresented categories. This approach helped to enhance the diversity of the minority class samples and balanced their influence during training. Additionally, we closely monitored the sample distribution to ensure that the model would not be biased toward categories with a higher sample count, which in turn improved the model’s ability to recognize different types of floating debris accurately.

During the data cleaning and screening process, we collected an initial dataset of over 10,000 images related to floating objects from multiple open-access image databases. Regarding dataset cleaning, the first step was to remove low-resolution images. Some low-resolution images may have become blurred due to uploading or multiple reuses; such low-resolution images are unlikely to appear in real-world applications. These images lack sufficient visual detail, which could negatively impact model learning and provide limited value for model generalization, making their removal necessary. Secondly, we adjusted non-representative categories. Certain categories had very low occurrence frequencies in real-world settings and often had extremely small sample sizes. Based on an analysis of river and lake conditions, these less representative categories were merged with similar categories to ensure the model’s broad applicability. To further improve data quality, we also focused on handling outliers during the data cleaning process. By analyzing the image samples, we identified and removed those with exceptionally poor quality, particularly low-resolution, blurry, or distorted images, ensuring they would not negatively affect the model training. Additionally, we applied several image enhancement techniques during preprocessing. First, noise reduction was performed on the images using filters, which helped to minimize noise interference and improve the model’s learning performance. To further enhance the images, contrast enhancement was applied to some images, making the floating debris more prominent and aiding the model in better feature extraction and classification tasks. These preprocessing steps ensured the dataset’s representativeness and quality, laying a solid foundation for the subsequent model training.

**Fig. 1:**
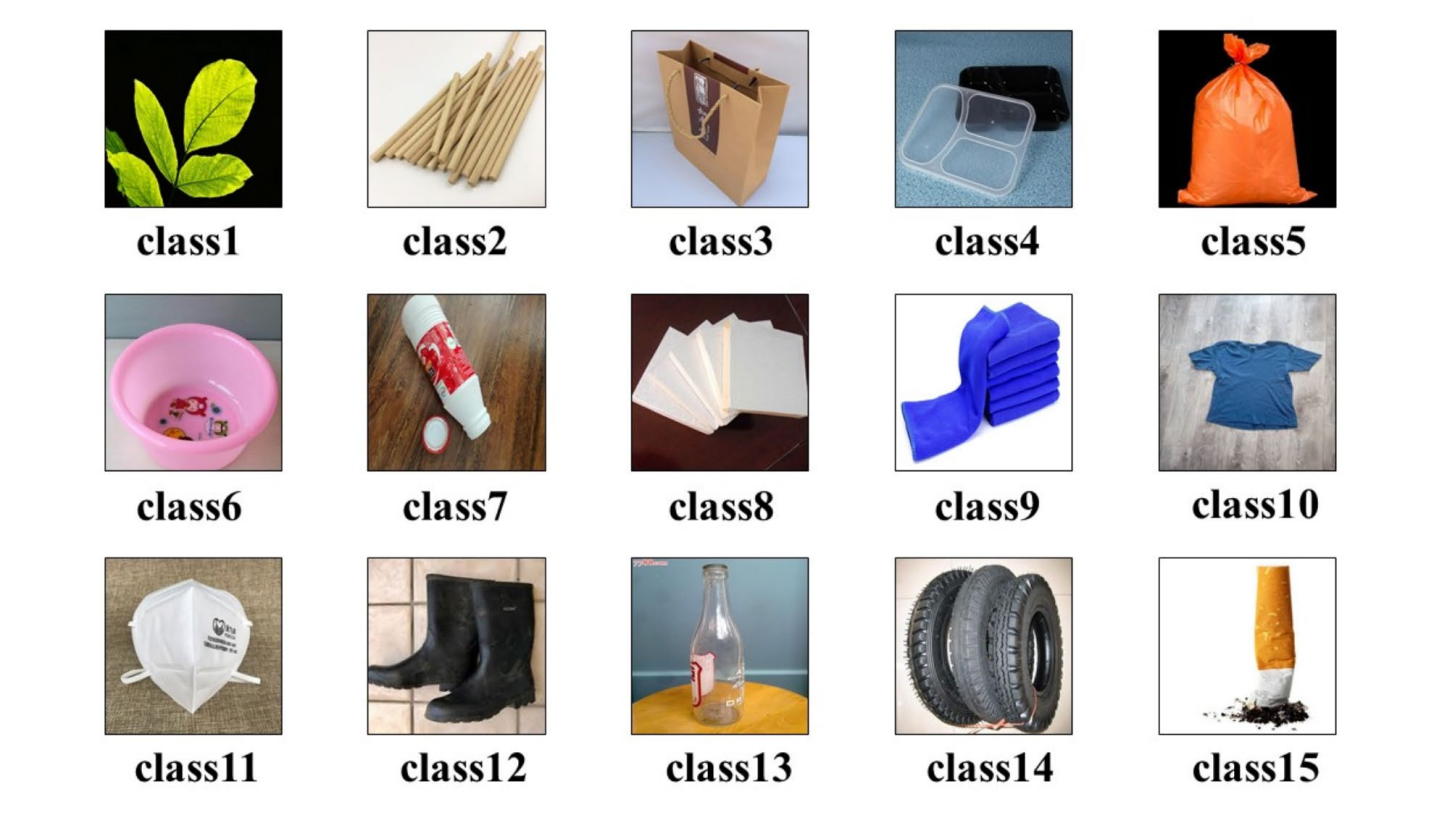
Images of a water surface floating object dataset with 15 categories.

These steps allowed the selected 15 categories to retain sufficient representativeness and reference value. Finally, 15 categories totaling 5707 images were screened as original images. The image categories are shown in Fig. 1.

In order to facilitate the extraction of models, all images undergo a screening process, after which they are numbered and assigned labels. They are then placed in relative folders and the dataset is partitioned into training and validation sets in the ratio of 4:1. This results in a training set comprising 4566 images and a validation set comprising 1141 images.

The types of floats and number of images are shown in Table 1.

**Table 1:** Types of floating objects and the number of images.

Main group	Form	Label	Training set	Validation set	Sum of samples
Natural degradation	leave	class1	85	21	106
	branch	class2	151	38	189
	paper bag	class3	386	97	483
Plastic products	lunchbox	class4	114	28	142
	plastic bag	class5	306	76	382
	plastic basin	class6	173	43	216
	plastic bottle	class7	232	58	290
	foam panel	class8	320	80	400
Textile goods	towel	class9	256	64	320
	clothes	class10	689	172	861
	respirator	class11	144	36	180
	shoes	class12	541	135	676
Others	glass bottle	class13	611	153	764
	wheel	class14	462	115	577
	cigarettes	class15	97	24	121
Sum			4566	1141	5707

## Modeling and improvement

### Introduction of CNN

Convolutional Neural Network (CNN) is deep learning models designed to process data with a grid-like structure^24,25^. CNN is particularly effective at recognizing and processing visual information, and utilize supervised learning methods to learn visual patterns directly from raw image data^26,27^. The core idea of this network is to mimic the way the human visual system works by extracting features from images layer by layer to recognize and classify visual objects^28^. The network structure of the entity can be described as follows:

1) Convolutional Layer.

The convolutional layer is the fundamental component of CNN and is responsible for extracting local features from an image. In the convolutional layer, a set of learned kernels are employed to perform convolutional operations on the image^29^. Each kernel performs a convolution operation with a small region of the image to generate a feature map^18^. Mathematically, this process can be represented as:

1 $$f_{{ij}}^{{(l)}}={\text{ReLU}}\left( {\sum\limits_{k} {\sum\limits_{{m,n}} {W_{{mnk}}^{{(l)}}} } \cdot x_{{i+m,j+n,k}}^{{(l - 1)}}+{b^{(l)}}} \right)$$

where: $f_{{ij}}^{{(l)}}$ is the activation value at position (*i*,* j*) on the feature map of layer *l*, $W_{{mnk}}^{{(l)}}$ is the weight of the convolution kernel, $x_{{i+m,j+n,k}}^{{(l - 1)}}$ is the input of the previous layer, and ${b^{(l)}}$ is the bias term.

2) Pooling Layer.

The pooling layer follows the convolutional layer and is used to reduce the spatial dimensionality of the feature map, reducing the amount of computation and the number of parameters, thus controlling overfitting^30^. Common pooling operations include maximum pooling and average pooling^31^. For example, in maximum pooling, the maximum value is selected from a local region of the feature map as a representative of that region with the mathematical expression:

2 $$p_{{ij}}^{{(l)}}=\hbox{max} \left( {x_{{ab}}^{{(l - 1)}}} \right)$$

where: $p_{{ij}}^{{(l)}}$ is the output value at position (*i*,* j*) after pooling of the layer *l* and $x_{{ab}}^{{(l - 1)}}$ is the corresponding input value of the previous layer in the pooling window.

3) Fully connected layer.

After the spatial features of the image have been extracted by the convolutional and pooling layers, the fully connected layer is used to combine and classify these features at a high level^32^. In this layer, all the activation values of the previous layer are spread and connected into a vector, which is then passed to the next layer by means of full connectivity.

3 $${z^{(l)}}={W^{(l)}} \cdot {x^{(l - 1)}}+{b^{(l)}}$$

where: ${z^{(l)}}$ is the weighted input of layer *l*, ${W^{(l)}}$ and ${b^{(l)}}$ are the weights and bias of the layer, ${x^{(l - 1)}}$ is the output of the previous layer, which is obtained from that layer by means of an activation function.

4) Classification Layer.

The classification layer is usually located at the end of the network and is used to map the extracted features to the final output categories^33^. Using the Softmax function the output can be converted to a probability distribution that represents the probability that the input image belongs to each category.

4 $${\text{Softmax}}{(z)_i}=\frac{{{e^{{z_i}}}}}{{\sum\limits_{j} {{e^{{z_j}}}} }}$$

where: $z$ is the output of the last fully connected layer and ${\text{Softmax}}{(z)_i}$ is the probability that the input sample belongs to class *i*.

### VGG-16 model mechanism

The VGG-16 model was proposed by the Visual Geometry Group (VGG) at the University of Oxford, and is famous for its excellent performance in the 2014 ImageNet Challenge^34,35^. The “16” in VGG-16 refers to the fact that it consists of 16 weight layers, 13 of which are convolutional layers and 3 of which are fully connected layers. The core design concept of the VGG-16 model is to use repeated convolutional kernels of the same size and a maximum pooling layer to improve feature extraction by increasing the depth of the network^36^. This design simplifies the complexity of the network architecture while extracting higher level image features through the deep network structure^37^.

All convolutional layers in the model use a 3 × 3 convolutional kernel, a design that reduces the number of parameters, making the model deeper and more computationally efficient. Gradually, image features are extracted from low to high level by stacking multiple convolutional layers^38^. After every few convolutional layers, the model uses a 2 × 2 maximum pooling layer to reduce the spatial size of the feature map, thus reducing the number of parameters and computation in subsequent layers^39^.

VGG-16 ends with 3 fully connected layers, the first two fully connected layers have 4096 neurons each, and the last fully connected layer is tuned to the number of classes for the classification task, typically 1000 neurons (for ImageNet’s 1000-class classification task). The ReLU (Rectified Linear Unit) activation function is used after each convolutional and fully connected layer.

5 $${\text{ReLU}}(x)=\hbox{max} (0,x)=\left\{ {\begin{array}{*{20}{l}} 0&{{\text{if }}x<0} \\ x&{{\text{if }}x \geqslant 0} \end{array}} \right.$$

where: *x* denotes the vector of acceptance regions, this formula indicates that ReLU sets all negative input values to 0 and remains unchanged for non-negative input values, which helps to speed up the training of the model and solves the gradient vanishing problem.

In this study, we selected the VGG-16 model as the core architecture for feature extraction and classification, primarily due to its deep convolutional structure that enables multi-level feature extraction from images layer by layer. The feature selection process involves hierarchical feature extraction from lower to higher layers, with each convolutional layer progressively capturing local features in the images, such as edges, textures, and shapes, through different receptive fields. This hierarchical feature selection approach allows the model to better understand the visual characteristics of floating objects on the water surface, thus distinguishing different types of floating debris. A key advantage of the model lies in its multi-channel convolutional operations. The initial convolutional layers focus on extracting low-level features such as edges and simple shapes, while the deeper convolutional layers gradually capture more complex features, including textures, local patterns, and global shapes of objects. The feature selection progresses from simple to complex, and this layered approach ensures that the model can accurately differentiate essential features from noise when faced with complex images.

### Model improvement

The VGG-16 model was trained on ImageNet, and the model was migrated to a new model to help train the new model in order to improve training efficiency^40^. To enhance the performance of the VGG-16 model in the task of water surface floating object classification, specific improvements were made to the traditional VGG-16 model, resulting in the VGG16-15 model. The following are the improvement measures and their specific impact on model performance.

First, adjustments were made to the depth and number of convolutional layers. The traditional VGG-16 model consists of 13 convolutional layers and 3 fully connected layers, totaling 16 layers. To reduce the computational complexity and adapt to the classification needs of floating objects on the water surface, one convolutional layer was removed, simplifying the total number of layers to 15. This modification significantly reduces the model’s parameter count, making training more efficient on small datasets, avoiding feature redundancy, and thereby reducing the risk of overfitting. The simplification of convolutional layers also shortens training and inference time, enhancing the response speed of the VGG16-15 model in practical applications.

In terms of the number of channels in feature maps, the VGG16-15 model optimized the number of channels in some convolutional layers, especially in the middle layers, to reduce redundant channels in the feature maps. This adjustment significantly reduces computational overhead while retaining essential feature information. Reducing the number of channels not only streamlines the model but also improves its ability to distinguish between different categories of floating objects. The optimized VGG16-15 model achieves higher recognition accuracy and shows significant improvement in running speed compared to the traditional model.

**Fig. 2:**
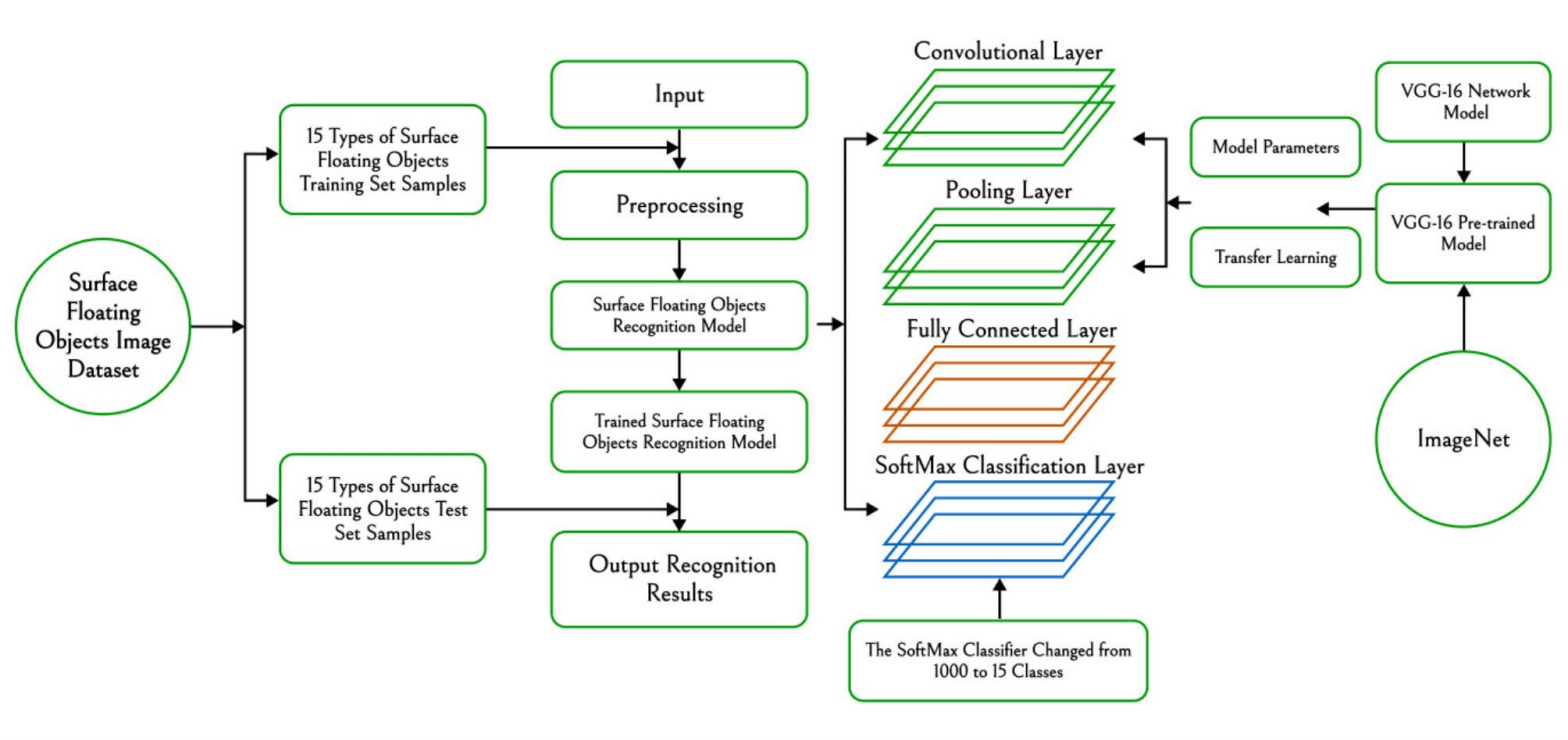
Framework of water surface floating object recognition model.

For the structure of the fully connected layers, the VGG16-15 model retained the convolutional layer weights pre-trained on ImageNet, while optimizing the fully connected layers. The original VGG-16 model had three fully connected layers with 4096 neurons each, whereas the floating object dataset in this study only contains 15 categories. Therefore, the last fully connected layer was modified to 15 neurons to match the output of 15 classification categories. In addition, the original Softmax classification layer was replaced with a new classification layer capable of outputting probabilities for 15 categories of floating objects on the water surface. This modification ensures that the model can accurately recognize categories in this study’s classification task, making it valuable for floating object classification scenarios. The framework of water surface floating object recognition model is shown in Fig. 2.

Although the VGG-16 model performs well in traditional image classification tasks, it presents several limitations in the classification of floating objects on water surfaces. First, due to the deep architecture and large number of parameters, VGG-16 is prone to overfitting when trained on small datasets, resulting in weak generalization. This overfitting issue is particularly evident in floating object datasets, where labeled data available for real-world applications is often limited. Additionally, the original model exhibits feature redundancy in its higher convolutional and fully connected layers, which not only increases computational complexity but also extends training and inference times.

**Fig. 3:**
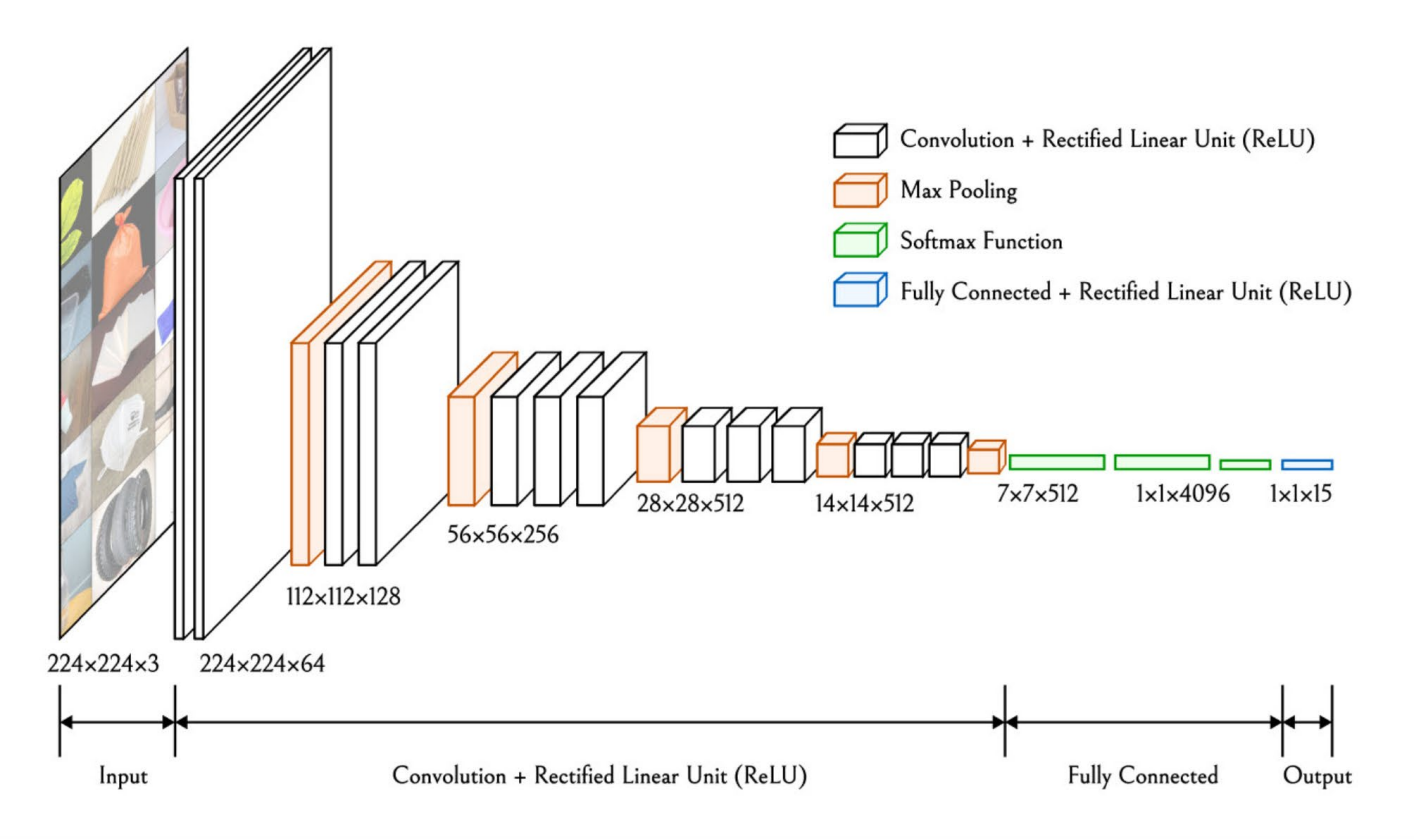
VGG16-15 convolutional neural network architecture.

To overcome these limitations, this study introduces several improvements to the VGG-16 model, adapting it to the specific needs of floating object classification. First, reducing the number of convolutional layers decreases the model’s parameter count, thus reducing computational complexity and enhancing the model’s generalization ability on small datasets. This adjustment effectively minimizes feature redundancy and mitigates overfitting. Secondly, the structure of the fully connected layers was optimized. The original fully connected layers contained a large number of neurons, leading to an excessive number of parameters. By reducing the number of neurons in these layers, the study significantly reduces overfitting risk. The optimized fully connected structure not only improves training efficiency but also enhances the model’s stability in the floating object classification task. Through these improvements, the VGG16-15 model has demonstrated significant enhancements in both generalization and real-time performance in floating object classification, validating the effectiveness of these adjustments in practical applications. The convolutional neural network architecture of the adjusted VGG16-15 model is shown in Fig. 3.

## Preprocessing and training process optimization

### Training environment

The model was trained based on MATLAB 2023b, which is a powerful high-level technical computing language and interactive environment developed by MathWorks. MATLAB has a built-in Deep Learning Toolbox (DLT) which provides a set of functions for designing, training and implementing deep neural networks. The processor used in this study is Intel Core i5 and CUDA is used for GPU acceleration to improve training efficiency^41^.

### Preprocess

In the training preprocessing phase, the dataset underwent a series of standardized processing steps to ensure data consistency and enhance model performance. First, a thorough cleaning of the dataset was conducted to remove any low-resolution images that lacked sufficient visual detail. Additionally, any mislabeled or irrelevant images were removed to maintain the dataset’s integrity and relevance to the classification task. Following the cleaning process, all images were resized and standardized to a resolution of 224 × 224 pixels, matching the input requirements of the VGG-16 model^42,43^. This resizing ensures that images maintain a consistent spatial structure across the dataset, allowing the convolutional layers to learn spatial hierarchies more effectively without being affected by variations in original image dimensions. This step is critical for ensuring that all data inputs have uniform dimensions, which reduces computational discrepancies during batch processing and improves overall training efficiency^44^.

To further enhance the model’s convergence and stability during training, each image was normalized by scaling pixel values to a [0, 1] range. In addition, the mean pixel value of the dataset was subtracted from each image, effectively centering the data distribution around zero. This normalization not only reduces the variance within the dataset but also accelerates the gradient descent process by preventing large fluctuations in weight updates, thereby ensuring a more stable convergence. These preprocessing steps collectively contribute to the model’s ability to learn robust and transferable features from the dataset, ultimately improving both the training efficiency and the accuracy of the VGG16-15 model in floating object classification tasks.

### Training optimization strategy

Training optimization strategies play a crucial role in the development of deep learning models, and appropriate strategies can not only improve the generalization ability of the model, but also accelerate the training process and improve the stability and efficiency of model training. Two main training optimization strategies are adopted in this study: data enhancement strategy and learning rate decay and early stopping strategy.

1) Data Enhancement Strategy.

In order to increase the generalization ability of the model and avoid overfitting, data augmentation techniques were employed in this study. The benefit of data augmentation is that it greatly improves the diversity of training samples and provides more varied data contexts, which in turn makes the model more capable of recognizing unseen data. During each round of training, images were processed with various augmentation techniques, including random rotation angles, random X-axis scaling, random Y-axis scaling, random X-axis panning, and random Y-axis panning. These transformations simulate different shooting angles and scale variations, increasing the diversity of the training data. Specifically, random rotation was applied by rotating images within a ± 30° range, which allowed the model to adapt to variations in the orientation of floating objects on the water surface, simulating the diverse angles at which floating debris may appear in real-world conditions. Next, random scaling was applied along both the X and Y axes to ensure the model could recognize objects of varying sizes, thereby improving its ability to detect floating debris with significant size differences. Additionally, random translation was applied by shifting images by ± 20%, simulating positional changes due to variations in camera angle. This enhanced the model’s ability to adapt to positional variations. Furthermore, random cropping helped the model focus on partial objects, improving its ability to recognize local features, especially when floating debris is partially occluded. These data augmentation techniques enabled the model to learn richer features, enhancing its generalization ability across various environments and significantly improving classification performance.

2) Learning rate decay and early stopping strategy.

Learning rate decay is a strategy to adjust the learning rate, which can gradually reduce the learning rate during the training process to help the model adjust the weights more finely and avoid excessive oscillations when minimizing the loss function. Combined with the early stopping strategy, i.e., stopping training when the performance on the validation set is no longer improving, it can prevent overfitting and save computational resources. The combination of these two strategies can find the optimal learning rate at different stages of training and stop training when the model performance stops improving, thus ensuring that the model reaches the optimal state. The model automatically adapts to the optimal learning rate, so there is no need to consider the effect of the learning rate on the model performance, and there is no need to design a learning rate control group.

### Model test and analysis

#### Training evaluation indicator

In model training, training accuracy and validation accuracy are important metrics for evaluating model performance. Training accuracy is the performance of the model on the training dataset. It measures how accurately the model predicts known data during training, and a high training accuracy usually means that the model learns the features of the training data well.

6 $${\text{Training Accuracy}}=\frac{{{N_{{\text{correct train}}}}}}{{{N_{{\text{total train}}}}}} \times 100\%$$

where: ${N_{{\text{correct val}}}}$ is the number of samples correctly predicted on the training set and ${N_{{\text{total val}}}}$ is the total number of samples in the training set. The training accuracy reflects the model’s ability to learn from the training data.

Validation accuracy is the performance of the model on an independent data set that was not used for training (validation set). This metric is used to assess the model’s ability to generalize to unseen data, and a high validation accuracy indicates that the model performs well on unseen data.

7 $${\text{Validation Accuracy}}=\frac{{{N_{{\text{correct val}}}}}}{{{N_{{\text{total val}}}}}} \times 100\%$$

where: ${N_{{\text{correct val}}}}$ is the number of samples correctly predicted on the training set and ${N_{{\text{total val}}}}$ is the total number of samples in the training set. The training accuracy reflects the model’s ability to learn from the training data.

The loss function is a key function used to evaluate the performance of a model, and its value reflects the error or inaccuracy of the model’s predictions. The goal of model training is to minimize the loss function by tuning the parameters. For the VGG-16 model, the loss function is shown as follows:

8 $${\text{Cross-Entropy}}= - \frac{1}{N}\sum\limits_{{i=1}}^{N} {\sum\limits_{{c=1}}^{C} {{y_{i,c}}} } \log ({\hat {y}_{i,c}})$$

where: *N* is the total number of samples, C is the total number of categories, ${\text{ }}{y_{i,c}}$ indicates whether the sample *i* belongs to the category *c*, ${\text{ }}{\widehat {y}_{i,c}}$ and is the probability that the model predicts that the sample *i* belongs to the category *c*.

## Hyperparametric tuning

In order to explore the effects of epoch and batch size on the model performance, and at the same time, to determine the best set of hyperparameters. An initial learning rate of 0.001 and a learning rate change factor of 0.5 are selected, and different epochs and batch sizes are set to train and verify the characteristics of various types of water surface floaters, and analyze the effects of the above parameters on the classification effect of the model. The model classification and recognition performance before and after model improvement and before and after data enhancement is evaluated by accuracy, precision, recall and F1 score.

1) The effect of different epochs on the model.

**Fig. 4:**
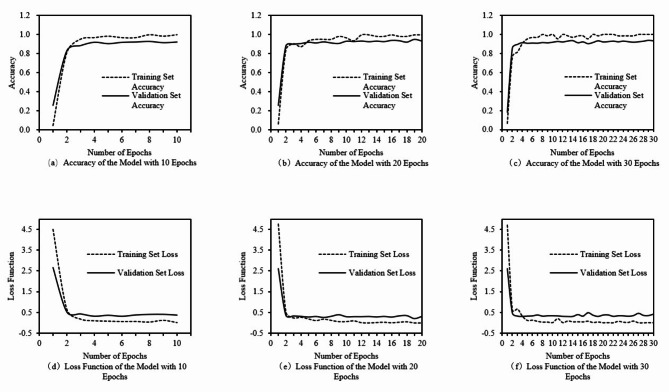
Accuracy and loss function of the model at different epochs.

Epochs refers to the number of times the model learns the entire dataset, and a epoch of 5 means that the model traverses the entire dataset 5 times and then stops training. A small number of epochs may prevent the model from learning the complete class features resulting in low accuracy, while a large number of epochs may produce overfitting, making the model less accurate on the validation dataset, so choosing the appropriate number of epochs is important for model performance. The number of epochs for the VGG16-15 model was set to 10, 20, and 30, respectively, with a fixed batch size of 64 to explore the appropriate number of iterations. At this time, the loss function change and accuracy change curves of the training and validation sets of the VGG16-15 model are shown in Fig. 4.

In Fig. 4, as the number of epochs continues to increase, the model learns more and more deeply about the data set, the model’s training accuracy and validation accuracy show an upward trend, and the values of the loss function for the training and validation sets gradually decrease. With the increasing number of epochs, the growth of the model’s training and validation set accuracies slows down, and gradually converges and remains stable.

When the number of epochs is 10, the model does not reach the ideal state of feature learning, although there is a certain degree of accuracy, but the fluctuation is larger, and there is no complete convergence; when the number of epochs is 20, the model reaches the ideal state of feature learning, the training accuracy of the model tends to be close to 100%, and the model validation accuracy fluctuates at a high level, which indicates that the model is more stable at this time and does not have overfitting. When the number of epochs is 30, the training accuracy of the model tends to be close to 100%, but the accuracy of the validation set is not significantly improved compared to the number of epochs is 20, and the value of the loss function is high. Therefore, choosing the number of epochs as 20 can ensure the model convergence, the model’s classification accuracy is high, and the model performance is good, and it also avoids the occurrence of overfitting, and the model stability is higher.

2) The effect of batch size on the model.

Batch size refers to the number of samples used in each iteration when training the neural network. Larger batches require more memory because more data need to be loaded and processed at one time, and too large a batch may not be efficiently processed with limited resources; smaller batches may lead to frequent and unstable gradient updates during model training. Therefore, an appropriate batch size is not only conducive to improving the training efficiency of the model, but also improves the generalization ability of the model, thus obtaining the best model performance. The number of iteration rounds is fixed at 20, and the batch sizes of 32, 64, and 128 are set for training, respectively, to obtain the accuracy and loss function changes of the corresponding training validation sets.

**Fig. 5:**
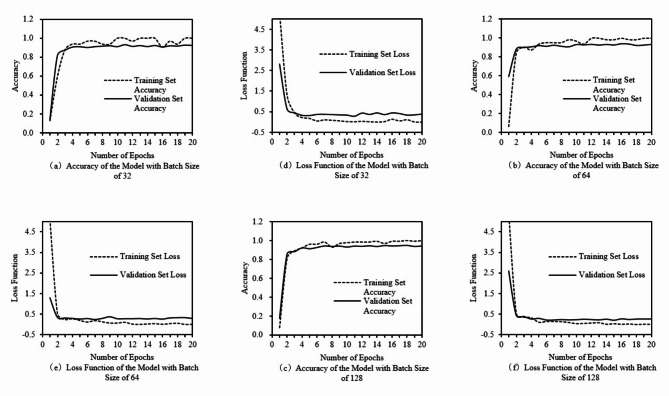
Accuracy and loss function of the model under different batch sizes.

The detailed data of the three training sessions are obtained and calculated to get the specific values of average verification accuracy, average verification loss function and the time required for training as shown in Table 2:

**Table 2:** Validation accuracy, loss function, and training time of the model under different batch sizes.

Batch size	Epochs	Validation accuracy (average)	Validation loss (average)	Training time (min)
32	20	0.87	0.50	37.2
64	20	0.90	0.35	42.7
128	20	0.89	0.37	197.9

From Fig. 5; Table 2, it can be seen that the validation accuracy and the validation loss function values do not reach the ideal state when the batch size is 32; while when the batch size is 128, although the validation accuracy and the validation loss function values are more ideal, the time required for training is much longer than expected; and when the batch size is 64, the model’s convergence is fast, and at the same time the model validation accuracy is also kept at a relatively high level, and the model’s fluctuations are smaller and therefore more stable. Therefore, a batch size of 64 is ideal.

By adjusting the number of iteration rounds and the batch size of the two hyperparameters, the neural network with the increasing number of iteration rounds, the training accuracy and verification accuracy of the model gradually increase, and the value of the loss function also decreases and gradually tends to 0. By adjusting the two hyperparameters of the two hyperparameters, it is found that: when the number of iteration rounds is 20, and the batch size of the batch size is 64, it can obtain a better recognition effect of the model, and the verification set of the accuracy is as high as 95.1%, and the loss function can reach 95.1%, and the loss function value is as low as 64. It can reach 95.1%, and the loss function value is only 0.1954, which also shows that the model has a good performance, can have a high accuracy in the case of low loss function value, and can well meet the task of recognizing and classifying floating objects on the surface of the water.

### Model recognition performance analysis

In order to systematically and completely evaluate the performance of the model, four metrics are chosen to be evaluated using Accuracy, Precision, Recall and F1 Score, which are commonly used evaluation metrics for deep learning models, and they are mainly computed through confusion matrices. These metrics combine different aspects of the model’s performance in a classification task.

**Fig. 6:**
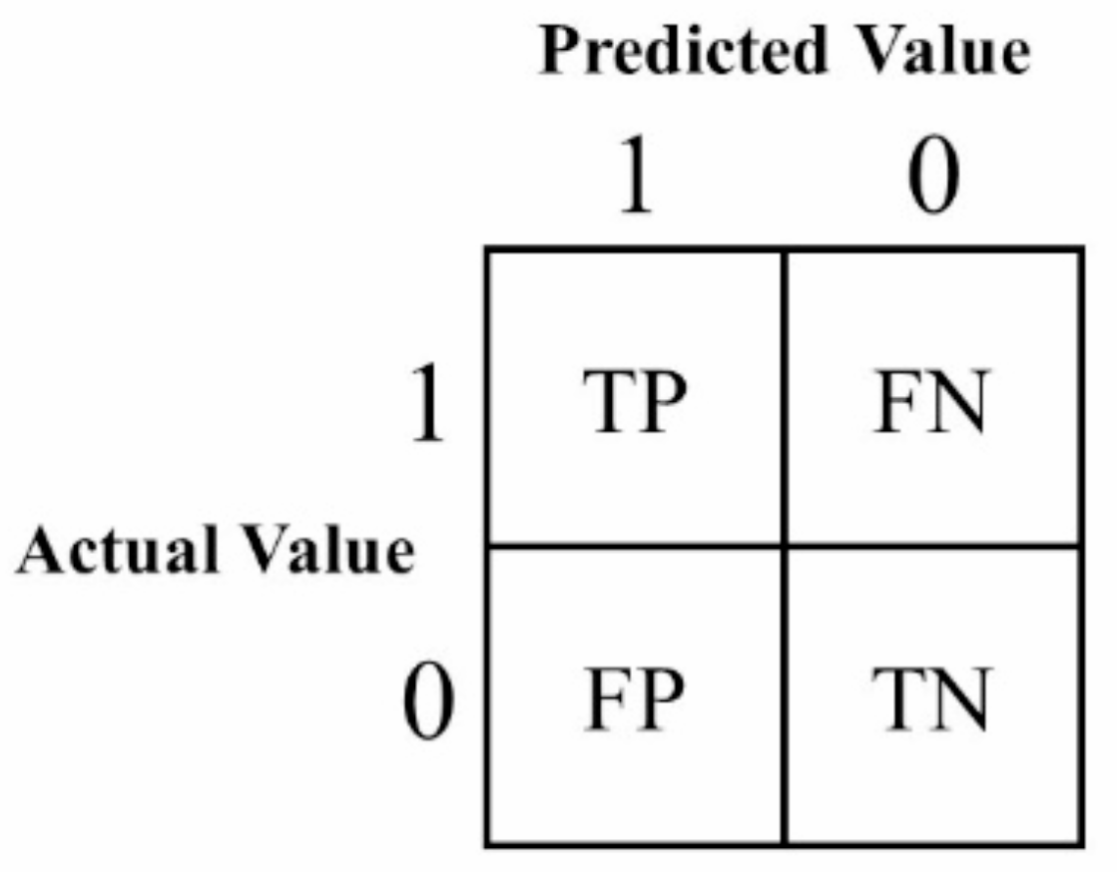
Confusion matrix diagram.

The confusion matrix is a table that is used to evaluate the performance of a classification algorithm. For binary classification problems, it consists of four components: true positive examples (TP), false negative examples (FN), false positive examples (FP) and true negative examples (TN). In a multi-categorization problem, this matrix will be extended to the predicted results between each category and the others. The relationship between the indicators FP, TP, FN and TN is shown in Fig. 6.

Accuracy is the most intuitive performance metric, indicating the ratio of the number of samples correctly classified by the model to the total number of samples, and this metric responds to the model’s overall ability to correctly classify samples.

9 $$A{\text{cc}}uracy=\frac{{TP+TN}}{{TP+TN+FP+FN}}$$

Precision measures the proportion of samples predicted by the classification model to be positively classified that are actually positively classified; this metric reflects the quality of the samples predicted by the model to be positively classified, with a high precision indicating that fewer negatively classified samples are misclassified as positively classified.

10 $$Precision=\frac{{TP}}{{TP+FP}}$$

Recall indicates the proportion of all samples that are actually positively classified that the model correctly predicts to be positively classified; this metric reflects the model’s ability to retrieve positively classified samples, with a high recall meaning that fewer positively classified samples are missed.

11 $$Recall=\frac{{TP}}{{TP+FN}}$$

F1 score is a reconciled average of precision and recall, and in order to provide a balance between the two, the metric is a combined evaluation of the model’s ability to accurately identify positive classes and its ability to retrieve positive classes, which is particularly applicable in the case of class imbalance.

12 $$F1=2 \times \frac{{Precision \times Recall}}{{Precision+Recall}}$$

To further explore the performance of the model and the advantages of data enhancement, the traditional VGG-16 model without data enhancement, the improved VGG16-15 model without data enhancement, and the improved VGG16-15 model with data enhancement were selected as controls. At the end of model training, the validation set is used as the test set to derive the precision rate of the corresponding trained models for the classification of the 15 categories for comparison, and the overall Accuracy, Precision, Recall and F1 Score of the models are also obtained as the indexes for evaluating the performance of the models.

**Fig. 7:**
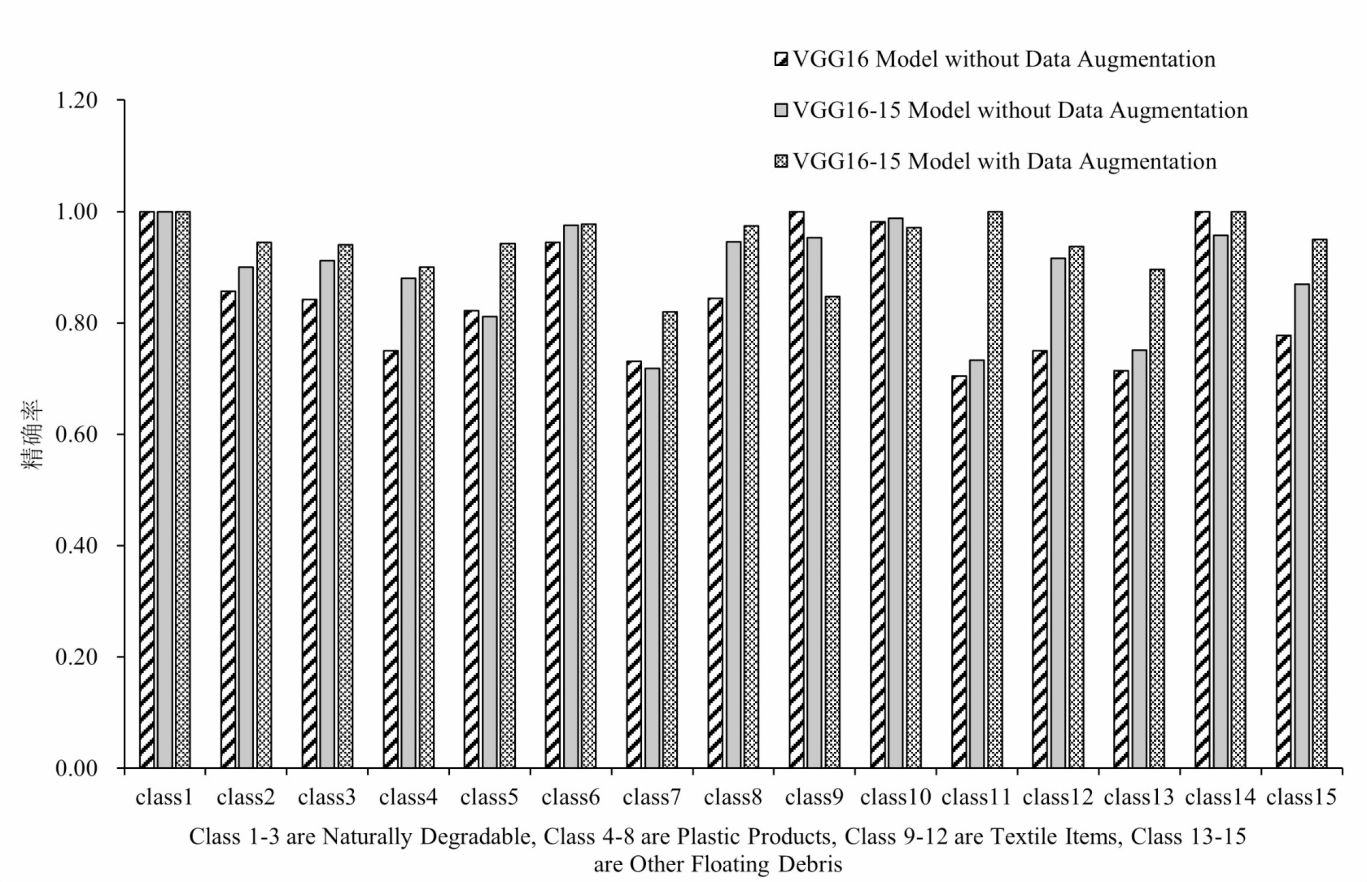
Recognition precision of traditional VGG16 model without data augmentation, VGG16-15 model without data augmentation, and VGG16-15 model with data augmentation.

The Fig. 7 shows the recognition accuracy of the three models for the 15 categories of floating objects on the water surface:

The overall assessment indicator data corresponding to the three models are shown in Table 3:

**Table 3:** Overall evaluation metrics for the three models.

Model type	Accuracy	Average precision	Average recall	Average F1 Score
Traditional VGG-16 model without data augmentation	83.77%	84.78%	78.39%	79.72%
VGG16-15 model without data augmentation	88.68%	88.75%	86.33%	86.97%
VGG16-15 model with data augmentation	93.86%	94.00%	92.38%	93.03%

The traditional VGG-16 model without data augmentation has an accuracy of 83.77%, an average precision of 84.78%, an average recall of 78.39%, and an average F1 score of 79.72%. Despite the high accuracy rate, the low average recall rate indicates that the model has some deficiencies in retrieving positive class samples. The VGG16-15 model without data enhancement has an accuracy of 88.68%, an average precision of 88.75%, an average recall of 86.33%, and an average F1 score of 86.97%. Compared with the traditional VGG-16 model, the VGG16-15 model shows significant improvement in all evaluation metrics, especially in the Recall and F1 score, indicating that the model does a better job in balancing the accuracy and completeness of the check. The VGG16-15 model with data augmentation has an accuracy of 93.86%, an average precision of 94.00%, an average recall of 92.38%, and an average F1 score of 93.03%. The model demonstrated the best performance, especially in terms of significant improvement in accuracy and average F1 score. The VGG16-15 model with data augmentation shows a 5.18% improvement in recognition accuracy over the VGG16-15 model without data augmentation, which demonstrates that data augmentation is crucial for improving model performance. The recognition accuracy of the VGG16-15 model with data enhancement is improved by 10.09% compared to the traditional VGG-16 model without data enhancement, and the recognition accuracy of the VGG16-15 model without data enhancement is also improved by 4.91% compared to the traditional VGG-16 model without data enhancement. This also indicates the good results of the Softmax classifier designed for the classification task with 15 classes of target objects.

The performance of the model on different categories is analyzed, and the model performs relatively well on most of the categories, with two categories, class7 and class9, where the recognition accuracy of the model is much lower than average. The water surface floating object category of class7 in the dataset is beverage bottles, whose features are more similar to glass bottles, and beverage bottles have a wide range of colors, types, and shapes, and the number of samples is not rich enough, so there may be a lack of learning, which leads to a lower recognition accuracy. The category of floating objects on the water surface corresponding to class9 in the dataset is towel, and its texture, shape and other features are similar to textile items such as clothes, which may be the reason for the low recognition accuracy. Subsequently, the model recognition accuracy can be improved by further enriching the sample data, cleaning the acquired dataset more deeply and other strategies.

### Few-shot classification test

To verify the adaptability and generalization ability of the fine-tuned model under conditions of limited sample data, we conducted a few-shot classification test. The objective of this test was to observe whether the fine-tuned VGG16-15 model demonstrates superior classification performance in a low-sample setting, further validating the effectiveness of fine-tuning for this specific task.

In this experiment, three categories (class3, class6, class9) were randomly selected from the 15 floating object categories, with only 3 images per category, resulting in a total of 9 images for the few-shot test set. The fine-tuned VGG16-15 model and the non-fine-tuned VGG-16 model were both evaluated on this few-shot test set to compare their classification accuracy. The experimental results are shown in Table 4.

**Table 4:** Comparison of classification accuracy between fine-tuned and non-fine-tuned models on few-shot test set.

Category	Number of Samples	Correctly Classified by Fine-Tuned Model(VGG16-15)	Correctly Classified by Non-Fine-Tuned Model(VGG16)
Class3	3	3	1
Class6	3	2	2
Class9	3	2	1
**Total**	9	7	4

The fine-tuned model correctly classified 7 out of 9 images, achieving an accuracy of 77.8%, whereas the non-fine-tuned model only correctly classified 4 images, with an accuracy of 44.4%. These results indicate that the fine-tuned VGG16-15 model has a stronger recognition capability in few-shot settings and can more accurately capture the features of the target categories. This demonstrates that the fine-tuning process effectively enhances the model’s generalization ability with limited sample data.

The above content demonstrates that the model performs well and meets the design requirements of the task. However, during actual deployment, the model may face additional challenges, such as limited computational power in embedded systems, which necessitates further research on compatibility with embedded systems and model efficiency. Moreover, environmental factors like weather changes and lighting conditions can also impact the model’s recognition accuracy. To address these challenges, future research could focus on developing lightweight models and exploring adaptive tuning based on deployment conditions, enhancing the model’s adaptability and robustness in dynamic environments, thus supporting broader applications in environmental monitoring and autonomous navigation.

## Conclusion

The recognition of surface floating objects is crucial for the navigation safety of unmanned vessels and the efficiency of garbage collection. In this paper, based on convolutional neural network and migration learning with the VGG-16 model, we design and train a recognition model for 15 types of water surface floating objects. It is verified that the model has good accuracy in recognizing floating objects on the surface of the water, and the types of floating objects can be expanded according to task needs. Future research directions could focus on exploring lighter and more efficient deep learning architectures suitable for embedded systems, enhancing the real-time recognition capability on unmanned platforms. Finally, developing and testing the model under different weather, lighting, and water conditions would further strengthen its robustness, supporting more extensive applications in environmental monitoring and autonomous navigation.

## Data Availability

The data supporting the findings of this study are available from the corresponding author upon reasonable request.
